# FROM KNOWLEDGE TO PRACTICE: TRAINING FAITH LEADERS IN AGING AND MENTAL HEALTH

**DOI:** 10.1093/geroni/igae098.3358

**Published:** 2024-12-31

**Authors:** Annalee Wilson, Bronwyn Keefe, Erin Emery-Tiburcio

**Affiliations:** Boston University, Boston, Massachusetts, United States; Boston University, Boston, Massachusetts, United States; Rush University, Chicago, Illinois, United States

## Abstract

Faith leaders can play a leading role in recognizing and supporting the mental health of their older congregants. Older adults attend faith services more frequently than members of other age groups and often turn to faith leaders for mental health support. To increase faith leaders’ knowledge and skills pertaining to mental health and aging, the Center for Aging and Disability Education and Research (CADER) developed an online course, Mental Health and Aging for Faith Leaders. In this course, participants learn to identify mental health conditions, to describe barriers to treatment, to identify services and resources available for treating mental health conditions in older adults, and to explain the role that faith leaders can play in supporting older adult mental health. CADER piloted the course with 44 faith leaders from Massachusetts (n=7) and Chicago, Illinois (n=37). Of the Chicago-based faith leaders, all except one indicated that they serve a congregation that is majority Black. Faith leaders showed significant improvements (p< 0.05) in all learning competencies from the pre-course competency self-assessment to the post-course competency self-assessment. When asked what changes they anticipate making after taking the course, faith leaders reported that they intended to make changes on the individual level (e.g., by continuing to learn more about aging and mental health); the interpersonal level (e.g., by identifying the signs of mental health concerns in their older congregants); the organizational level (e.g., by implementing mental health programming at their faith organizations); and at the societal level (e.g., by promoting awareness about mental health).

